# A TOR (target of rapamycin) and nutritional phosphoproteome of fission yeast reveals novel targets in networks conserved in humans

**DOI:** 10.1098/rsob.200405

**Published:** 2021-04-07

**Authors:** Lenka Halova, David Cobley, Mirita Franz-Wachtel, Tingting Wang, Kaitlin R. Morrison, Karsten Krug, Nicolas Nalpas, Boris Maček, Iain M. Hagan, Sean J. Humphrey, Janni Petersen

**Affiliations:** ^1^ Faculty of Life Sciences, University of Manchester, Oxford Road, Manchester M13 9PT, UK; ^2^ Proteome Center Tuebingen, University of Tuebingen, Auf der Morgenstelle 15, 72076 Tuebingen, Germany; ^3^ Flinders Health and Medical Research Institute, Flinders Centre for Innovation in Cancer, Flinders University, Adelaide, South Australia 5042, Australia; ^4^ Cancer Research UK Manchester Institute, Alderley Park, Macclesfield SK10 4TG, UK; ^5^ Charles Perkins Centre, School of Life and Environmental Sciences, The University of Sydney, Camperdown, New South Wales, Australia; ^6^ Nutrition and Metabolism, South Australia Health and Medical Research Institute, North Terrace, Adelaide, South Australia 5000, Australia

**Keywords:** TORC1, TORC2, nitrogen stress, phosphoproteome, fission yeast *Schizosaccharomyces pombe*, Byr1 MAPKK

## Abstract

Fluctuations in TOR, AMPK and MAP-kinase signalling maintain cellular homeostasis and coordinate growth and division with environmental context. We have applied quantitative, SILAC mass spectrometry to map TOR and nutrient-controlled signalling in the fission yeast *Schizosaccharomyces pombe*. Phosphorylation levels at more than 1000 sites were altered following nitrogen stress or Torin1 inhibition of the TORC1 and TORC2 networks that comprise TOR signalling. One hundred and thirty of these sites were regulated by both perturbations, and the majority of these (119) new targets have not previously been linked to either nutritional or TOR control in either yeasts or humans. Elimination of AMPK inhibition of TORC1, by removal of AMPK*α* (*ssp2::ura4^+^*), identified phosphosites where nitrogen stress-induced changes were independent of TOR control. Using a yeast strain with an ATP analogue-sensitized Cdc2 kinase, we excluded sites that were changed as an indirect consequence of mitotic control modulation by nitrogen stress or TOR signalling. Nutritional control of gene expression was reflected in multiple targets in RNA metabolism, while significant modulation of actin cytoskeletal components points to adaptations in morphogenesis and cell integrity networks. Reduced phosphorylation of the MAPKK Byr1, at a site whose human equivalent controls docking between MEK and ERK, prevented sexual differentiation when resources were sparse but not eliminated.

## Graphical abstract

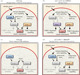

## 1. Introduction

All eukaryotic cells are exquisitely sensitive to changes in their external environments and constantly adapt their metabolism and rate of division to meet dynamic changes in nutrient availability. When nutrient supply is abundant, cells maintain high levels of protein synthesis to increase biomass and support cell division. By contrast, cell proliferation ceases in nutrient-starved conditions in order to conserve energy, maintain homeostasis and ensure survival [1]. In healthy animal tissue, cells proliferate with a remarkably consistent size at division, demonstrating tight coordination between cell growth and division within specific environmental contexts [2–4]. These controls are modified upon nutritional stress, in which resources are limited but not eliminated, to reduce cell size at division [5], thereby lowering demands on resources required for the growth of each cell generation. This coupling of nutrition and division in turn maintains proliferation, despite reduced nutrient availability.

Several distinct signalling pathways mediate nutrient sensing [1]. The highly conserved AMP-activated protein kinase (AMPK) and target of rapamycin (TOR) kinases are two major energy and nutrient sensors in eukaryotic cells [6]. Exposure to nutrient stress alters AMPK and TOR signalling to regulate cell growth and cell cycle progression through coordinated action of MAPK (mitogen-activated protein kinase) and CDK (cyclin-dependent kinase) signalling [2–4,7–9]. As this confluence of AMPK activation and TOR inhibition stimulates CDK control of size at division, it is hard to distinguish between the contributions of AMPK and TOR to CDK and cell-cycle-specific signalling. Fission yeast is an excellent model system in which to overcome this challenge, as all the signalling networks are highly conserved and yet can be genetically isolated from one another to support the independent study of these normally contiguous nutritional-sensing signalling networks.

TOR signalling comprises two structurally and functionally distinct multi-protein complexes that regulate the flux through distinct signalling networks to a multitude of targets. TOR kinases form TORC1 and TORC2 (TOR Complex 1 and 2), which are defined by unique subunits that are highly conserved across species; the protein Raptor defines mammalian TORC1 (mTORC1), while Rictor is uniquely found in mTORC2 [10]. In the fission yeast *Schizosaccharomyces pombe* model that is the focus of this study, Mip1 defines TORC1 and Ste20 defines TORC2 [11–13].

Closely reflecting the subtleties of nutrient modulation in human cells, nitrogen starvation and nitrogen stress elicit fundamentally different responses in fission yeast, even though it is the same nutrient that is being either partially (stress) or completely (starvation) depleted in the two distinct responses. We have previously shown how the imposition of nitrogen stress in fission yeast (defined here as a change from a good to a poor nitrogen source) transiently activates AMPK, which in turn inhibits TORC1 to accelerate mitotic entry, and cell division continues at reduced cell size [7–9,14]. By contrast, the inhibition of TORC1 by amino acid starvation promotes cell cycle exit, does not require AMPK [7] and so differs from the control of TORC1 by AMPK in response to nitrogen stress. While it is well established that both nitrogen stress and starvation induce autophagy, we have shown how the acceleration in mitotic entry in response to nitrogen stress is unaffected by an inability to activate autophagy in fission yeast [15]. Thus, the reduced cell growth and cell size at division in response to nitrogen stress is an entirely distinct response and does not arise from the induction of autophagy and ensuing amino acid sensing. Recently, we and others have highlighted parallels in human cells to the nitrogen stress response of yeasts, as a reduction in ammonia provision also activates AMPK and inhibits mTORC1 in human cells lines [7,14,16].

Although nitrogen stress reduces TORC1 activity, it has no impact upon TORC2 activity [7]. It is therefore reminiscent of the acute and specific inhibition of TORC1 signalling by rapamycin, that also promotes mitosis and cell division [9]. In contrast with the specific targeting of TORC1 by rapamycin, the ATP analogue Torin1 competitively inhibits the kinase activities of both the TORC1 and TORC2 complexes [17,18]. In striking parallels to the fission yeast controls, the enhanced impact of Torin1 on cell growth and proliferation in mammalian cells is mediated by inhibition of rapamycin-insensitive elements of TORC1 signalling, rather than an additive impact on both mTORC1 and mTORC2 activities [17]. Thus, compared to rapamycin, Torin1 is a more potent inhibitor of TORC1 and simultaneously inhibits TORC2 to completely ablate all TOR signalling. TOR inhibition through Torin1 treatment also promotes mitotic commitment and a reduction in cell size at division. We have shown how the degradation of the major negative regulator of mitosis, Wee1 kinase, plays a key role in TOR control of cell division in human cell lines [18].

Here, we applied SILAC and mass spectrometry-based phosphoproteomics to map fission yeast TOR and nitrogen sensing global signalling. To this end, we combined the ability to ablate TOR signalling with Torin 1, with ease to eliminate TORC1 inhibition upon nitrogen stress through deletion of the gene encoding AMPK kinase α subunit. We also block Cdc2 signalling to eliminate indirect phosphorylation changes that would arise from cell cycle fluctuations driven by the alterations in the rate of division that normally couple TOR and AMPK growth controls to division. These manipulations combined with comparisons with datasets from cell cycle studies [19,20] enabled us to assign specific changes in the phosphoproteome to distinct signalling networks. We illustrate the utility of the resulting map of the nutritional phosphoproteomic landscape through demonstrating that a reduction in the phosphorylation of the MAPK kinase Byr1 upon nitrogen stress attenuates the differentiation response to nitrogen starvation. As this phosphorylation site resides in the interaction motif between the analogous kinase (MEK) and its substrate (ERK) in human cells, we anticipate that the fission yeast database can be used to reveal similar parallels in less well-characterized areas of the nutritional landscape of human cells. In this regard, we report fluctuations in the phosphorylation status of conserved molecules that are yet to be linked to TOR and nutrient signalling in human cells.

## 2. Results

### 2.1. Extensive fluctuation in global phosphorylation in response to nitrogen stress and TOR inhibition

**Figure 1.**
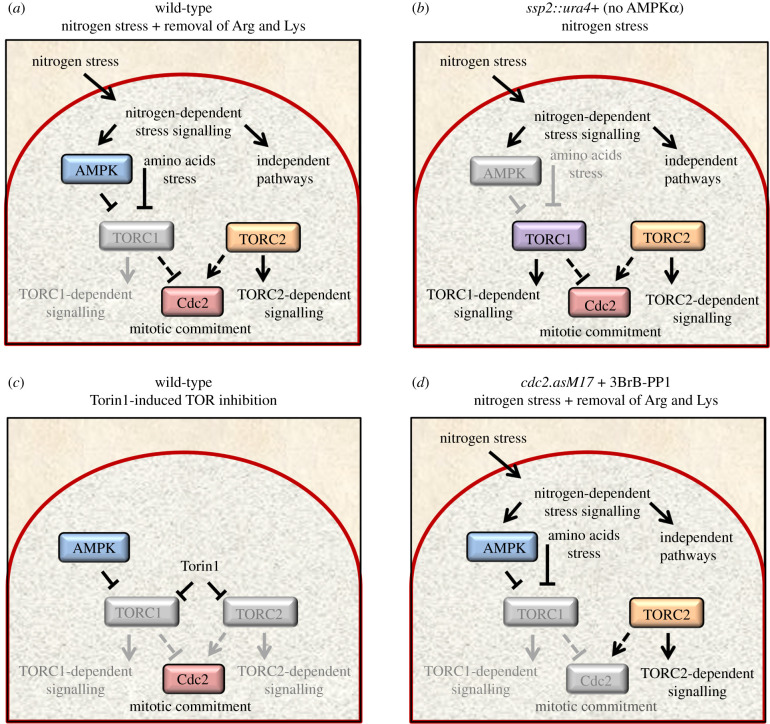
Overview of experimental perturbations. (*a*–*d*) Overview of strategy to identify nitrogen and TOR-regulated phosphorylation sites. Specific pathways that will be inhibited by the indicated perturbation are shown in grey for each panel. (*a*) Nitrogen stress: a change from a good (glutamate) to a poor (proline) nitrogen source, transiently activates AMPK, which, in turn, inhibits TORC1 to accelerate mitotic entry and cell division continues at reduced cell size. Nitrogen stress does not inhibit TORC2 [7]. Removal of arginine and lysine also generates amino acid stress. (*b*) The *ssp2::ura4+* AMPKα deletion strain ‘freezes’ nitrogen stress signalling prior to TORC1 inhibition and the ensuing increase in mitotic commitment that would otherwise arise from TORC1 inhibition. (*c*) Addition of Torin1 to glutamate grown cells blocks both TORC1 and TORC2 signalling. (*d*) To block mitotic commitment as we imposed nitrogen and amino acid stress, the ATP analogue 3BrB-PP1 was added to the *cdc2.asM17* mutant (CDK1) [21].

To identify potential TORC1- and TORC2-specific signalling pathways and to generate a comprehensive understanding of nitrogen-controlled signalling in fission yeast, four independent stable isotope labelling in cell culture cultured (SILAC) and mass spectrometry-based investigations were performed (figure 1). Three strains were exposed to nitrogen stress by altering the nitrogen source in the growth media from a good nitrogen source (glutamate) to a poor nitrogen source (proline): a wild-type strain, a strain in which the cyclin-dependent kinase Cdc2 could be inhibited upon the stress (*cdc2.asM17*) and a strain deleted for the AMPK*α* subunit (*ssp2::ura4^+^*). In addition, TOR signalling was inhibited in wild-type cells with Torin1.

Nitrogen stress activates AMPK, which in turn inhibits TORC1 [7]. Reduced TORC1 signalling activates Cdk1–Cyclin B (fission yeast Cdc2/Cdc13) to enhance mitosis and cell division [5,9,18]. As a consequence, alterations to phosphorylation identified when wild-type cultures are exposed to nitrogen stress combine nitrogen-specific, TORC1-specific and Cdc2/cell-cycle-specific regulations (figure 1*a*). We therefore exploited different genetic backgrounds, to determine the individual contribution of each signalling pathways to the complex nutrient response network. The AMPKα deletion strain was included in the analysis to ‘freeze’ signalling prior to TORC1 inhibition and the ensuing increase in mitotic commitment that would otherwise arise from TORC1 modulation (figure 1*b*) [7]. Phosphorylation sites that change following nitrogen stress in this AMPKα deletion strain are therefore likely to represent nitrogen stress-dependent signalling events that sit upstream and are independent of AMPK signalling. In contrast with nitrogen stress, which reduces TORC1 signalling alone and has no impact on TORC2 flux [7] (figure 1*a*), Torin1 efficiently inhibits both TORC1 and TORC2 signalling (figure 1*c*). Comparing phosphorylation sites that fluctuate after Torin1 treatment with the sites regulated by nitrogen stress enables us to identify sites that are affected by Torin1 alone and so are likely to be regulated by TORC2. This will therefore identify the subset of sites that are regulated by both nitrogen and Torin1, which are likely to be subject to TORC1 control (figure 1). Finally, altered phosphorylation in response to TORC1 inhibition by either nitrogen stress or Torin1 will include an over-representation of mitotic sites that are not a core part of nitrogen and stress signalling. Rather, these sites are a secondary consequence of the induction of mitosis by the nitrogen stress. Therefore, to eliminate spikes of phosphorylation that would accompany the increase in cells progressing through mitosis and be over-represented in the datasets at the 30 min sampling point, we included the conditional *cdc2.asM17* mutant (CDK1) [21] so that we could use the ATP analogue 3BrB-PP1 to block mitotic commitment as we imposed nitrogen stress (figure 1*d*).

The individual strains were cultured with ‘medium’ arginine and lysine labelled amino acids for a minimum of eight divisions. This ensured the complete incorporation of labelled amino acids into the phosphoproteome (electronic supplementary material, figure S1a). Arginine and lysine are both very potent activators of TORC1 signalling. In mammalian cells, both arginine and lysine activate mTORC1 via the lysosomal sensor SLC38A9 [22]. The imposition of nitrogen stress to unlabelled wild-type cells, by changing the nitrogen source from glutamate to proline, promotes a burst of cell division that peaks 60 min after the media switch (electronic supplementary material, figure S1b) [9]. Importantly, we needed to omit exogenous arginine and lysine (used to label to the proteome) from proline containing media upon nutrient stress of the wild-type and *cdc2.asM17* strains (see details for the AMPK*α ssp2::ura4^+^* deletion below), as mitotic commitment was not dramatically advanced when exogenous arginine and lysine remained in the media after nitrogen stress (electronic supplementary material, figure S1b). Therefore, the nitrogen stress of wild-type and *cdc2.asM17* cells we report here identifies changes to phosphorylation which can be considered to be part of a more general nitrogen-stress sensing network, that incorporates responses to arginine and lysine starvation alongside a shift in nitrogen quality (figure 1*a,d*).

AMPK is essential for nitrogen stress (glutamate to proline)-induced TORC1 inhibition [7]. However, the additional removal of arginine and lysine used here to expose wild-type and *cdc2.asM17* strains to nitrogen stress will inhibit TORC1 independently of the nitrogen stress response we want to monitor (figure 1*a*). Therefore, to gain insight into nitrogen-controlled signalling and avoid fluctuations in sites that are regulated by amino acid provision, modulation of TORC1 and the cell-cycle-regulated sites downstream of Cdc2, TORC1 activity was preserved by maintaining arginine and lysine in the proline ‘stress-inducing’ media used for the analysis with the *ssp2::ura4^+^* (AMPKα) deletion strain. Preservation of Maf1 phosphorylation after 60 min of nitrogen stress in *ssp2::ura4^+^*, contrasts with the Maf1 dephosphorylation that occurs within 15 min in the response of wild-type cells to the stress to indicate that both TORC1 and TORC2 (as monitored by Gad8.S546 phosphorylation) activity was maintained in these *ssp2::ura4^+^* cultures, despite the imposition of nitrogen stress (electronic supplementary material, figure S1c–f). To achieve inhibitor-based ablation of TOR signalling, Torin1 was added to glutamate grown ‘medium’ labelled cells. In one parallel ‘light’ control culture, cells were filtered back into glutamate medium that included arginine and lysine, whereas in the control for the Torin1 treatment, we simply added the solvent used for Torin1 (DMSO).

We prepared total protein extracts 30 min after filtration of cultures or the addition of Torin1, in order to quantitatively identify fluctuating phosphorylation sites by mass spectrometry. This 30 min time point was chosen in all experiments as the time point to chart the fluctuations, because we have previously shown how TORC1 readouts such as Maf1 dephosphorylation are readily detected at this time point after either nutrient stress or Torin1 addition [7,18].

We performed two biological repeats of each of: Torin1 treatment, nitrogen stress of wild-type cells and nitrogen stress of *cdc2.asM17* to which 3BrB-PP1 was added at the time of media change and the appropriate controls to which the phosphorylation proteome of the manipulated cultures would be compared. Thus, for the glutamate to proline shifts of the wild-type *ssp2::ura4^+^* and *cdc2.asM17* cultures, the reference cultures to which the comparison was made were filtered from glutamate medium, into fresh, prewarmed, glutamate medium, which, for the *cdc2.asM17* alone, contained solvent control for 3BrB-PP1. For the Torin1-treated cultures, the medium was not changed, but the control reference culture was the one to which we added the equivalent volume of the DMSO solvent used to deliver Torin 1.

The burst of mitosis and cell division (measured from aliquots of same cultures used for phosphoproteomics) peaked at 60 min after the change in conditions in both biological duplicates of Torin1-treated and nitrogen-stressed wild-type cultures (electronic supplementary material, figure S2a,b). This indicated that the treatments successfully elicited the responses we extensively characterized previously [7,9,18]. The cessation of cell division (judged by the frequency of septation that accompanies cytokinesis at the end of cell division) upon Cdc2 inhibition, 90 min after the shift, indicated that there had been an effective block to mitotic commitment at the preceding 30 min time point when samples were taken (electronic supplementary material, figure S2c). The absence of an impact of nitrogen stress upon the frequency of cell division in the *ssp2::ura4^+^* (electronic supplementary material, figure S2d) is consistent with the block to AMPK repression of TORC1 activity in this background (electronic supplementary material, figure S1d). Thus, in the ssp*2::ura4^+^* culture, it is clear that TORC1 activity persists despite the nitrogen stress that would normally elicit the response seen in the wild-type cells in electronic supplementary material, figure S1B.

In total, we identified 8077 phosphorylation sites with a localization probability of greater than 0.75 on 1920 unique fission yeast proteins (table 1; electronic supplementary material, data table S1). For each biological duplicate of the Torin1-treated culture and nitrogen-stressed wild-type culture, phosphorylation at approximately 200 sites changed at least twofold (*p* ≤ 0.05). Whereas phosphorylation fluctuated at 350 sites when cell division was arrested during the nitrogen stress by Cdc2 inhibition in the *cdc2.asM17* background*.* Finally, the changes were reduced from 250 to 150 sites when AMPK signalling repression of TORC1 was blocked in the nitrogen-stressed *ssp2::ura4^+^* strain (table 1; electronic supplementary material, data tables S2–S8).

**Table 1.** Summary of all phosphorylation sites identified in this study. Quantitative, SILAC and mass spectrometry-based analysis of global TOR and nitrogen-controlled signalling in fission yeast. A total of 8077 phosphorylation sites were identified with a localization probability of greater than 0.75 on 1920 unique fission yeast proteins. The number of specific phosphorylation sites and proteins identified for each perturbation is listed. Specific information for all sites is listed in electronic supplementary material, data tables S1–S8.

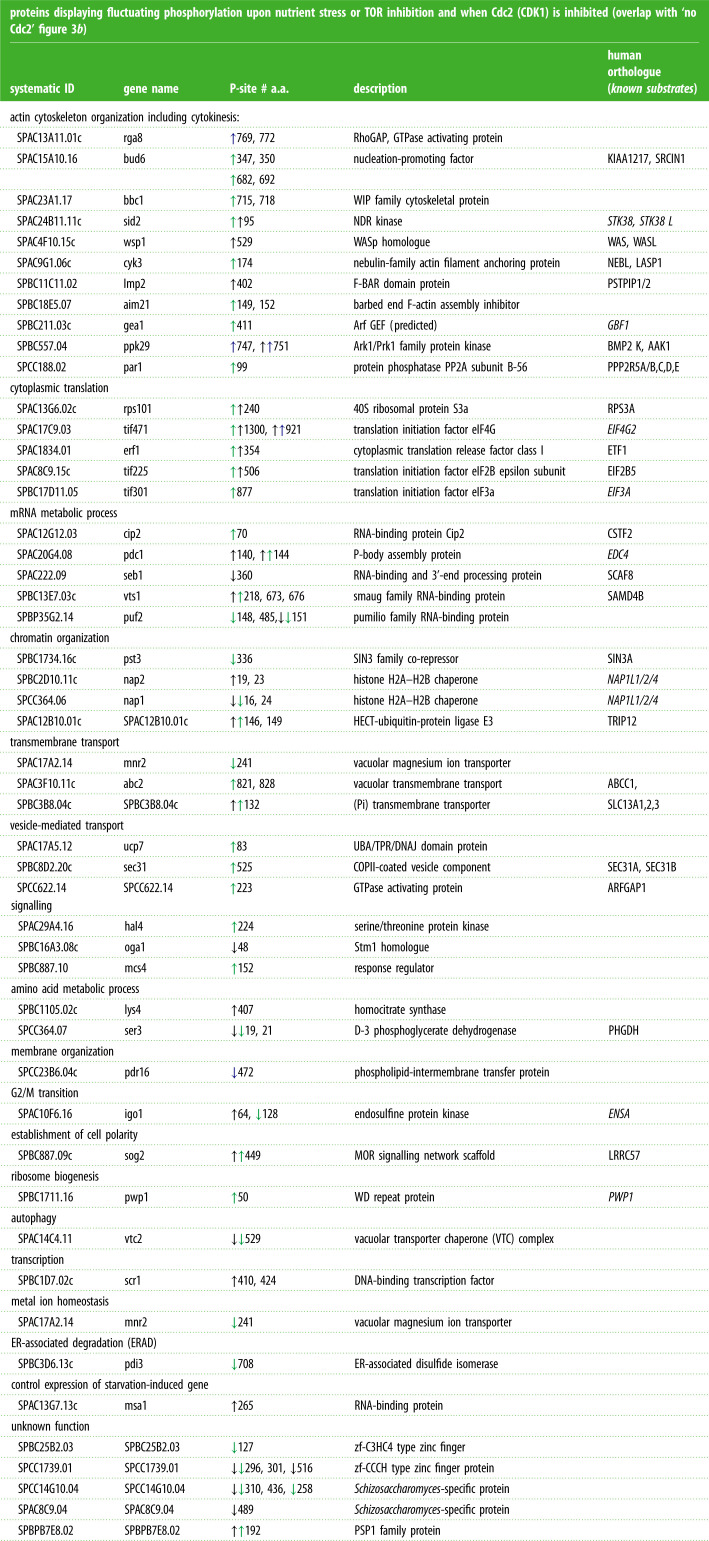

**Figure 2.**
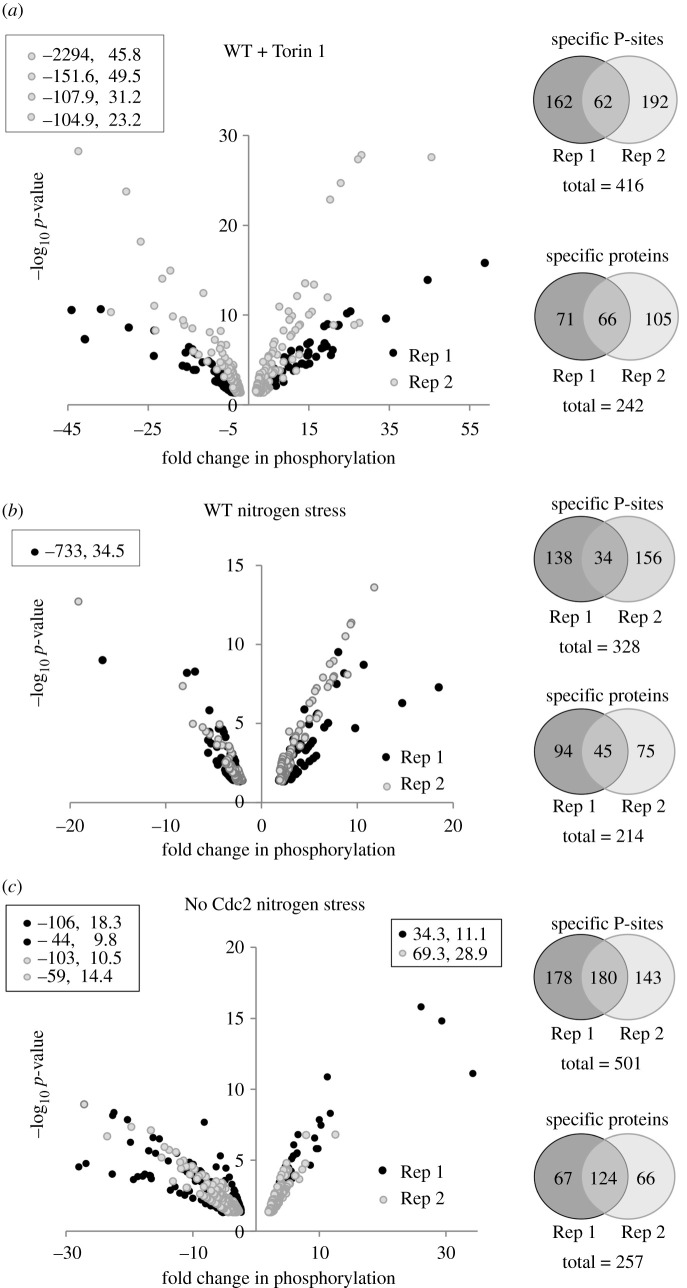
Similar levels of enrichment and reduction in phosphorylation level throughout the phosphoproteomes. (*a*–*c*) Significant changes to phosphorylation of specific phosphorylation sites greater than or equal to 2*×*, *p* ≤ 0.05 after 30 min perturbation compared to unstressed control are plotted as volcano plots for the individual perturbations indicated, data points listed within boxes falls outside plotted ranges. (Right) Venn diagrams illustrate the number of re-identifications of the same phosphorylation site or protein. (*a*,*b*) For each biological duplicate of the Torin1-treated culture and nitrogen-stressed wild-type culture, phosphorylation at approximately 200 sites changed. Twenty-seven per cent and 20% of the sites, respectively, were identified again in the repeat experiment. (*c*) Phosphorylation at 350 sites for each biological duplicate fluctuated when cell division was arrested during the nitrogen stress by Cdc2 inhibition in the *cdc2.asM17* background and 50% were identified again in the repeat experiment. Specific information for all sites is listed in electronic supplementary material, data tables S2–S8.

Recurrent identification of changes at the same site of phosphorylation in both repeats of Torin1 treatment or nitrogen stress of wild-type was seen at 27% and 20% of the sites, respectively, whereas the frequency at which the same sites were found to change in the consecutive experiment was 50% for the nitrogen-stressed *cdc2.asM17* cultures (figure 2). However, it is important to note that the *cdc2.asM17* experiments were measured on a newer instrument under different conditions. Interestingly, the frequency at which a change in phosphorylation anywhere on a specific protein was identified in the repeat of the perturbations was higher than repeat observation of a change in phosphorylation at a specific site: 50% for Torin1 treatment of wild-type, 33% for nitrogen stress of wild-type and 65% for combined nitrogen stress and Cdc2 inhibition of *cdc2.asM17.* This indicates that many of the target proteins are regulated by fluctuations in phosphorylation on multiple sites (figure 2). We combined the data from biological duplicates for all further analysis of fluctuations in phosphorylation in any given perturbation.

**Figure 3.**
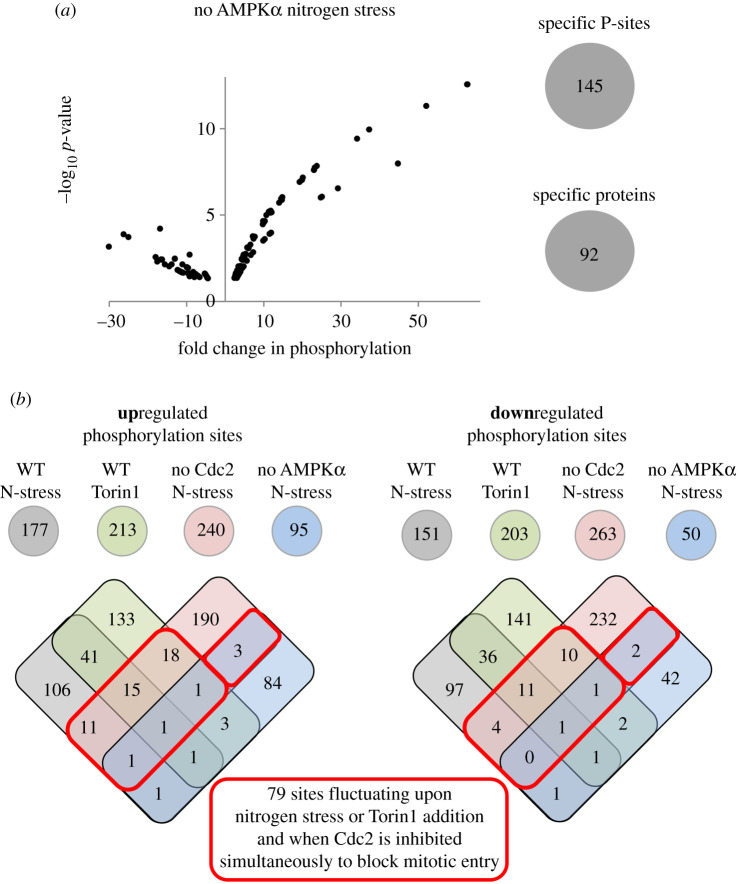
Sites regulated by nitrogen stress in the absence of AMPK activation and TORC1 inhibition and overview of overlap between individual datasets. (*a*) Significant changes to sites of specific phosphorylation greater than or equal to 2*×*, *p* ≤ 0.05 after 30 min nitrogen stress (change of nitrogen source from glutamate to proline) compared to unstressed control of the *ssp2::ura4^+^* (AMPK α) deletion strain is plotted as volcano plots. (Left) The number of specific phosphorylation sites and proteins identified are indicated. Specific information for all sites are listed in electronic supplementary material, data table S6. (*b*) The number of sites at which phosphorylation was either enhanced (upregulated) or reduced (downregulated) for each perturbation dataset. Venn diagrams were used to compare all specific phosphorylation sites identified for each perturbation. The numbers of re-identification of the same specific phosphorylation site in different datasets are shown in regions of overlap. Red boxes highlight specific phosphorylation sites that are regulated by nitrogen stress or Torin1 and also when Cdc2 is simultaneously inhibited to block mitotic entry. Specific information for these 79 sites is listed in table 2 and electronic supplementary material, data table S11.

In summary, when we compared the fluctuations in phosphorylation status seen in nitrogen-stressed and Torin1-treated cultures with the appropriate controls, we noted changes in the status of phosphorylation of at least twofold on: 416 sites (242 proteins) when signalling was inhibited with Torin1, 328 sites (214 proteins) for nitrogen stress of wild-type, 501 sites (257 proteins) when the burst in cell division that normally accompanies nitrogen stress was blocked by inhibition of *cdc2.asM17* and 145 sites (92 proteins) when AMPK signalling to TORC1 in the nitrogen stress response is blocked by *ssp2::ura4^+^* (figures 2 and 3*a*; electronic supplementary material, data tables S9 and S10). A striking feature of the datasets is that, even though the adaptations to the nutrient context monitored are driven by protein kinases, the frequency at which phosphorylation was seen to increase was roughly matched by the number of times we saw a decline in phosphorylation. Clearly, this could stem from the cessation of kinase signalling, as well as the stimulation of protein phosphatase activity.

Such extensive changes of phosphorylation sites on many proteins following nitrogen stress or TOR inhibition are consistent with the breadth of cellular changes required to adapt to nutritional change. Almost three-quarters of protein-coding genes in fission yeast have orthologues in human cells, and 70% of proteins identified across all four perturbation groups we performed are conserved in human cells (electronic supplementary material, data tables S9 and S10—this table lists human and *S. cerevisiae* orthologues of all proteins mentioned below), highlighting the potential utility of these data for studying both yeast and human metabolism.

### 2.2. Extensive novelty within the TOR and nutritional phosphoproteome

Before proceeding with further analysis, datasets were checked for positive controls in the form of changes in the phosphorylation status of molecules that have already been linked to TOR and nutritional signalling. Phosphorylation at sites on more than 80 orthologues of known substrates of TOR and nutrient signalling changed in our datasets, providing strong validation of our approach (electronic supplementary material, table S1). Importantly, phosphorylation on three proteins—Gad8 (AKT homologue), Gcn2 and Sty1 (P38, JNK homologue)—that we have previously linked to the response to nitrogen stress [9] fluctuated in response to nitrogen stress. The ribosomal protein S6 (Rps601) is a substrate of TORC1 signalling [23], Rps601 was dephosphorylated in response to nitrogen stress (electronic supplementary material, data table S10).

A number of previous studies have used phosphoproteomics-based techniques to map TOR signalling in budding yeast and mammalian cell lines [24–27]. The mammalian studies employed three different mTOR inhibitors, including the ATP competitive inhibitors Ku-0063794 and Torin1, and an allosteric inhibitor, Rapamycin on HEK293, and both normal and TSC−/− MEFs and Raptor and Rictor-deficient MEFs alongside the mapping of the response of MCF7 breast cancer cells to ammonia and rapamycin [28]. An extensive number of the proteins identified in our present study were also identified as conserved effectors of TOR and nitrogen signalling in these previous studies (electronic supplementary material, table S1). Importantly, 119 proteins of our targets conserved from yeast to humans are, to the best of our knowledge, novel targets in TOR or nitrogen-dependent signalling (electronic supplementary material, tables S2–S4).

### 2.3. Fluctuations in actin organization, RNA metabolism, vesicle and transmembrane transport and chromatin organization in the cell-cycle-independent dataset

To distinguish Torin1 and nitrogen-regulated phosphorylation events from potential secondary sites that are regulated due to Cdc2 (Cdk1–Cyclin B) activation, we compared specific phosphorylation sites that fluctuated upon the four individual perturbations (figure 3*b*). A similar number of specific phosphorylation sites were either up- or downregulated in all four perturbation groups. Of these, 79 sites still fluctuated when mitosis and cell division were blocked by inhibition of Cdc2 (figure 3*b*; electronic supplementary material, data table S11). The majority of these proteins have not previously been linked to TOR and nitrogen signalling (table 2). However, known components were also identified in this group (table 2, shown in italic), including the PP2A-B55 inhibitor Igo1 (ENSA), on which serine 64 phosphorylation has previously been shown to increase upon nitrogen stress to block PP2A-B55 activity [27,29]. Interestingly, a second site on Igo1 serine 128 is downregulated in response to Torin1 treatment in our data (table 2). Scr1 is a well-established target of AMPK signalling in fission yeast [30].

**Table 2.** A wide variety of biological processes are regulated independently of mitotic commitment. List of proteins displaying fluctuating phosphorylation upon nitrogen stress or TOR inhibition and when Cdc2 is inhibited (the same phosphorylation sites highlighted by ‘red boxes’ in figure 3*b*). Up or downregulation of the specific phosphorylation sites, along with human orthologue and GO-term mapping for the specific proteins are indicated. The specific residue regulated a.a. = amino acid is indicated. Up, or down pointing arrows indicate enhancement or reduction in phosphorylation, respectively. The colour code indicates the specific perturbation for which the site was identified; black, nitrogen stress of wild-type; blue, nitrogen stress of *ssp2::ura4^+^*; green, Torin1 addition to wild-type. All of the listed sites fluctuate upon nitrogen stress of *cdc2.asM17.* Sites identified in nitrogen stress cultures of: ↓ WT ↓ AMPK or cultures added ↓ Torin1. All sites fluctuate in nitrogen stress cultures of no Cdc2.

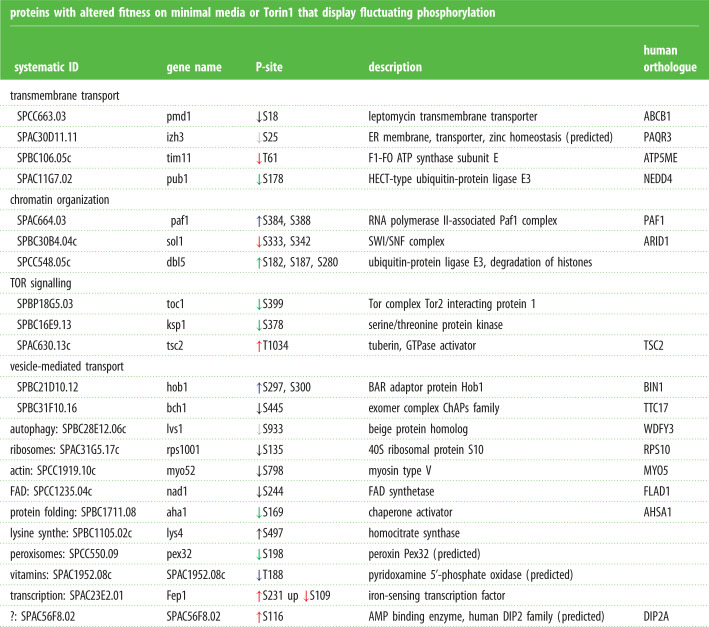

GO-term mapping also identified a wide variety of biological processes that have previously been associated with TOR and nitrogen signalling (table 2). Actin cytoskeleton organization, RNA metabolic process (including translation), transmembrane and vesicle-mediated transport as well as chromatin organization were among the processes with most regulators identified, suggesting that a significant number of proteins modulate these processes in response to environmental stress independently of cell cycle progression.

### 2.4. Nitrogen stress without AMPK activation and TORC1 inhibition invokes phosphorylation modulation of the actin cytoskeleton, cell polarity, transport and ribosome biogenesis among others

**Figure 4.**
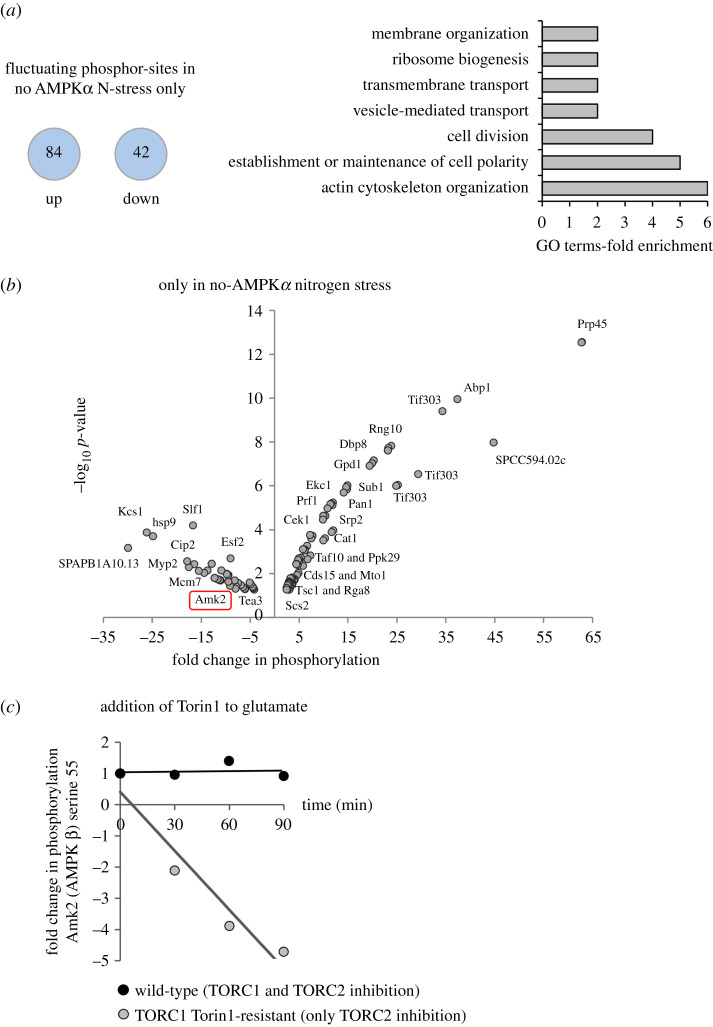
Phosphorylation sites changing upon nitrogen stress are enriched on proteins regulating the actin cytoskeleton, transport and ribosome biogenesis. (*a*) The number of specific phosphorylation sites that are up or downregulated upon nitrogen stress of *ssp2::ura4^+^* is shown. GO-term analysis (Princeton GOTermMapper) indicated which biological processed these proteins have been classified as participating in. (*b*) Specific changes to phosphorylation of specific phosphorylation sites only identified following nitrogen–stress of *ssp2::ura4^+^* (greater than or equal to 2*×*, *p* ≤ 0.05) are plotted on a volcano plots with examples of specific proteins labelled. The red box highlights the AMPK β subunit Amk2 which is analysed further in (*c*). (*c*) Small pilot time-resolved mass spec analysis of Fold change to Amk2 serine 55 phosphorylation compared to DMSO controls, when Torin1 is added to wild-type or a TORC1-torin1-resistant mutant. Specific information for all sites are listed in electronic supplementary material, data tables S12 and S13.

Because TORC1 activity and the frequency of cell division remained unchanged upon nitrogen stress of the *ssp2::ura4^+^* deletion strain (electronic supplementary material, figures S1d and S2d) [7], fluctuations in phosphorylation in this strain upon the imposition of nitrogen stress identified molecules that sit beyond the reach of AMPK and TOR control (figure 1*b*). A comparison between the datasets of all four individual datasets revealed sites at which phosphorylation fluctuated only in the nitrogen stress of *ssp2::ura4^+^* (figures 3*b* and 4*a*,*b*; electronic supplementary material, data table S12). GO-term analysis revealed a strong enrichment for proteins regulating the actin cytoskeleton and cell polarity, including Abp1, Pan1, Rga8, Tea3 Ppk29 and Myp2 (figure 4*b*; electronic supplementary material, data table S12). Vesicle and transmembrane transport, membrane organization and ribosome organization were also over-represented. While not enriched, a number of additional proteins involved in RNA regulation were identified, including Prp45, Srp2, Prp10, Cip2 Cwf2 and cwf21 regulating mRNA splicing, the translation factor Tif303 (EIF3c) and the Taf10 transcription factor component of the SAGA complex (figure 4*b*; electronic supplementary material, data table S12). These findings suggest that significant numbers of proteins involved in actin cytoskeleton organization, transport and RNA metabolism are modified in response to nitrogen stress by AMPK and TOR-independent signalling pathways.

One striking fluctuation in phosphorylation in the *ssp2::ura4^+^* dataset was the suppression of phosphorylation at serine 55 of the AMPK β subunit Amk2 (figure 4*b*; electronic supplementary material, data table S12), suggesting that the AMPK complex itself is regulated, in support of our previous findings [7]. Interestingly, in a pilot time-resolved SILAC phosphoproteome analysis, we also identified dephosphorylation of serine 55 of Amk2 (figure 4*c*; electronic supplementary material, data table S13) when TORC2, but not TORC1, was inhibited in a Torin1-resistant TORC1 mutant [18]. This suggests that Amk2 S55 is downregulated following environmental stress when TORC1 activity remains unchanged.

### 2.5. Comparison of Torin1 and nitrogen stress perturbations identifies potential TORC2, TORC1 and nitrogen-regulated phosphorylation sites

The comparison of phosphorylation sites that fluctuated in the four individual treatment groups identified many sites specific to each group (figure 3*b*). This may represent the true nature of signalling as, in contrast with nitrogen stress, the ATP competitive inhibitor Torin1 inhibits both TORC1 and TORC2 signalling (figure 1*a*,*c*). Alternatively, it may represent a technical limitation of this analysis. Importantly, 31 of the 77 fluctuating phosphorylation sites that were either up, or down, regulated in both Torin1 and nutrient stress of wild-type cells (figure 3*b*), but not regulated in the other two groups, were absent from the *cdc2.asM17* dataset all together (electronic supplementary material, data table S1—this table list all sites identified for all four perturbation, so information about re-identification for each perturbation are listed here). Hence, for these sites, we can state that they are regulated by either nitrogen or Torin1, but it is not possible to determine whether they represent true nitrogen and TOR-regulated sites, or change as a consequence of enhanced commitment to mitosis.

**Figure 5.**
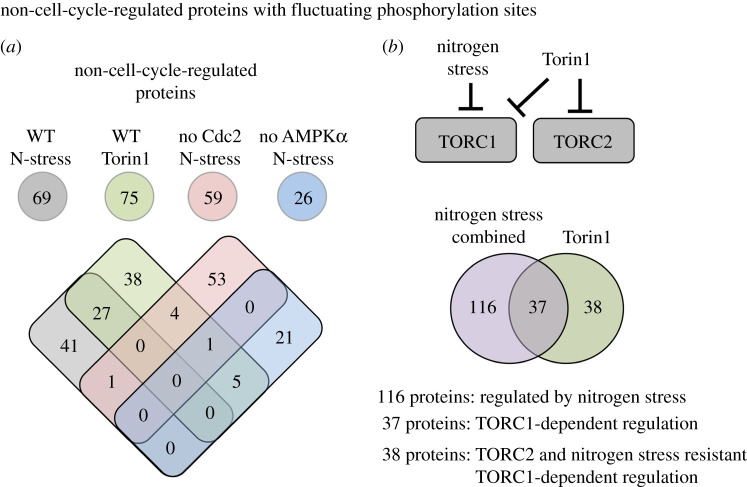
Prediction of likely nitrogen, TORC1 or TORC2 dependencies for ‘non-cell-cycle-regulated’ proteins. (*a*) All significantly nitrogen and Torin1-regulated substrates were compared to published datasets, that included information about cell cycle regulation and substrates of mitotic kinases, to identify ‘non-cell-cycle-regulated’ proteins with fluctuating phosphorylation sites among our datasets. The Venn diagram compares the overlap of proteins with fluctuating phosphorylation sites from each perturbation. (*b*) The upper images illustrate the reasoning behind grouping proteins into three groups of potential: nitrogen-regulated, TORC1-dependent and TORC2-dependent regulations. All ‘non-cell-cycle’ substrates regulated by nitrogen (from nitrogen stress of wild-type, *ssp2::ura4^+^ and cdc2.asM17)* were grouped in the lower Venn diagram to compare these with all Torin1-regulated substrates.

**Figure 6.**
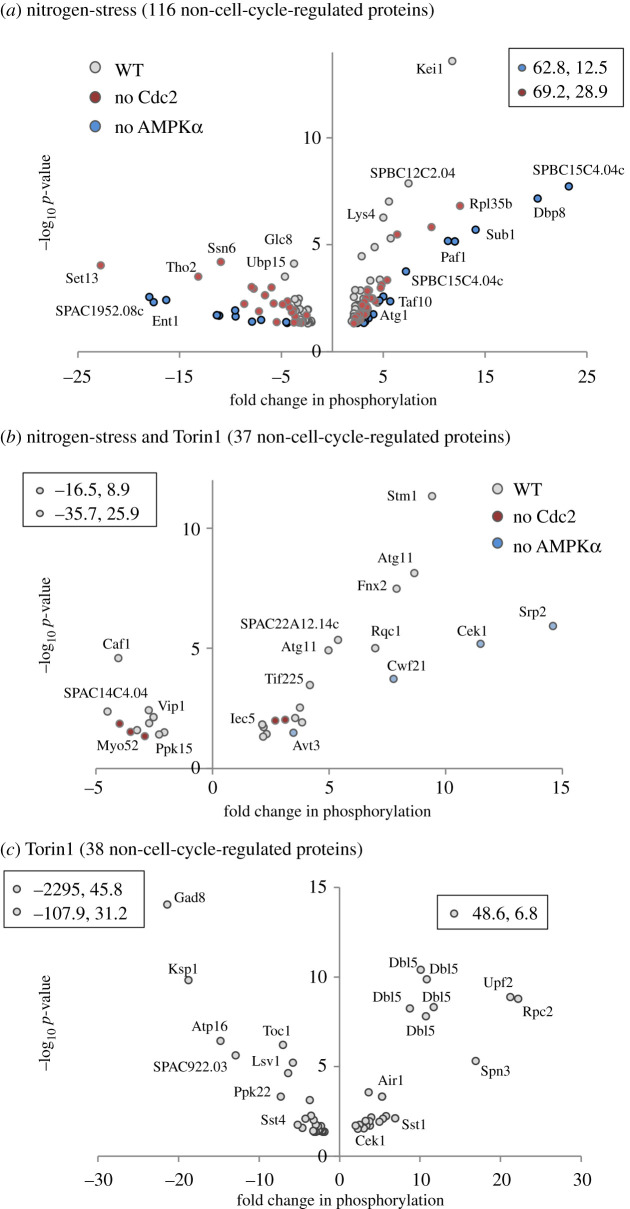
Volcano plots of specific phosphorylation sites likely to be regulated by nitrogen, TORC1 or TORC2. (*a*–*c*) Specific changes to phosphorylation of ‘non-cell-cycle’-regulated proteins, belonging to the three groups identified in figure 5*b*: (*a*) nitrogen-stress, (*b*) TORC1-dependent, (*c*) Torin1-dependent regulation; residues greater than or equal to 2*×*, *p* ≤ 0.05 are plotted as volcano plots with perturbation from which they were identified (grey, nitrogen stress of wild-type; red, nitrogen stress of *cdc2.asM17*; blue, nitrogen stress of *ssp2::ura4^+^*) and examples of specific proteins indicated. Data points listed within boxes falls outside plotted ranges. Specific information for all sites is listed in electronic supplementary material, data tables S14–S16.

To probe further into the contribution of mitotic phosphorylation to the datasets, we took advantage of published detailed analysis of Cdc2 and mitotic-specific phosphorylation regulation of the fission yeast proteome [19,20,31,32]. To pinpoint proteins in our datasets not previously identified as being substrates of Cdc2 or the mitotic kinase Polo and Aurora kinases Plo1 and Ark1, we compared these published datasets with all four perturbations to highlight ‘non-cell-cycle’-regulated substrates. Twenty-six proteins from nutrient stress of *ssp2::ura4*^+^ and approximately 70 proteins from each of the three other perturbations belonged to this group (figure 5*a*). Proteins regulated by both Torin1 and nitrogen-stress represent potential TORC1-regulated substrates. By contrast, sites changing in response to Torin1 inhibition are potential TORC2 substrates (figure 5*b*). For further analysis, all ‘non-cell-cycle’ proteins regulated by nitrogen stress (wild-type, *cdc2.asM17* and *ssp2::ura4*^+^ combined) were grouped together and compared with the Torin1-specific group (figure 5*b*; electronic supplementary material, data tables S14–S16). This identified around 40 potential TORC1 or TORC2-specific substrates alongside 116 proteins that were regulated by nitrogen stress. An equal number of specific phosphorylation sites were either up or downregulated in all three groups (figure 6). Importantly, positive controls were present in all three groups. The nitrogen stress group contained Atg1 (Ulk1), a known target of nutrient stress-induced autophagy [33]; the Paf1 complex, previously shown to be regulated by nitrogen stress [34]; and the SAGA complex, of which Taf10 is regulated by nitrogen starvation [35] (figure 6*a*; electronic supplementary material, data table S14). In the Torin1 and nitrogen stress co-regulated group, the PP2A-B55 regulating kinase Cek1 is a well-established downstream target of TORC1 in fission yeast [29], and Atg11 was previously identified as a target downstream of TORC1 in *S. cereviase* [36] (figure 6*b*; electronic supplementary material, data table S15). Finally, from the Torin1 only group, Gad8 (AKT) is a known substrate of TORC2 (figure 6*c*; electronic supplementary material, data table S16).

**Figure 7.**
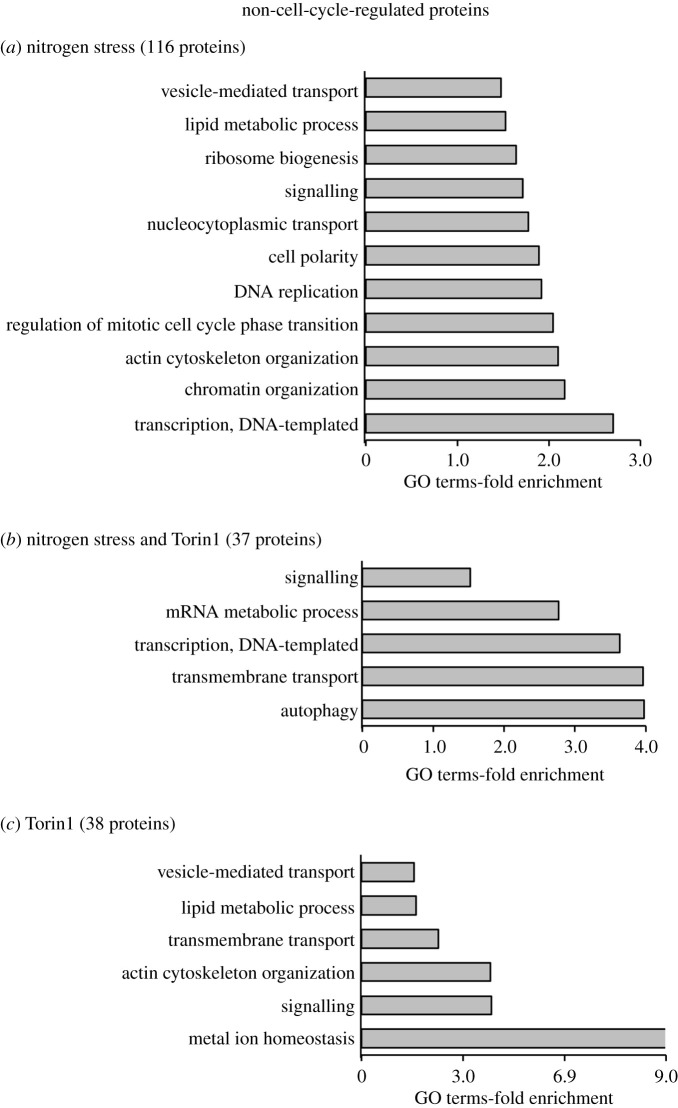
Go-term enrichments for proteins likely to be regulated by nitrogen, TORC1 or TORC2. (*a*–*c*) GO-term analysis (Princeton GOTermMapper) to identify biological function of ‘non-cell-cycle’-regulated proteins, belonging to the three groups identified in figure 5*b* ((*a*) nitrogen-stress, (*b*) TORC1-dependent, (*c*) Torin1-dependent regulation) with greater than or equal to 2*×*, *p* ≤ 0.05 fluctuating phosphorylation sites.

GO-term analysis of targets enriched in the nitrogen stress group revealed transcription, chromatin organization, actin cytoskeleton organization, transport and ribosome biogenesis among others (figure 7*a*). The well-established TORC1-regulated processes of autophagy, transmembrane transport, transcription and mRNA metabolic processes were enriched in the group regulated by both nitrogen stress and Torin1 treatment (figure 7*b*). Metal ion homeostasis is highly enriched in the set of targets where changes were only identified in the Torin1-treated dataset, that includes TORC2-specific candidates. This dataset also included strong representation from actin cytoskeletal organization, transmembrane transport and lipid metabolism (figure 7*c*). Consistently, TORC2 is a well-established regulator of actin, lipids [37] and transmembrane transport of glucose [38,39]; however, the potential link between TORC2 and transporters of metal ions is new.

### 2.6. Functional validation of novel substrates of TOR and nitrogen signalling

**Figure 8.**
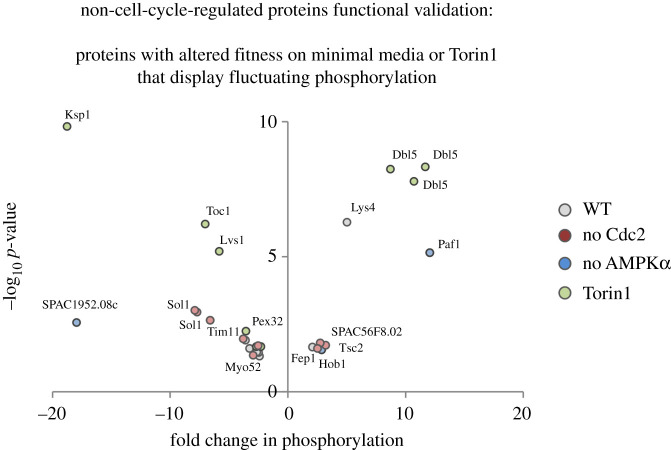
Functional validation for proteins likely to be regulated by nitrogen, TORC1 or TORC2. ‘Non-cell-cycle’ substrates belonging to the three group identified in figure 5*b* ((*a*) nitrogen-stress, (*b*) TORC1-dependent, (*c*) Torin1-dependent regulation) were compared to our published list of proteins for which the corresponding deletion strain which lacks the phosphorylated target is required for normal fitness in response to nitrogen stress or Torin1 treatment. The volcano plot illustrates significant change to phosphorylation greater than or equal to 2*×*, *p* ≤ 0.05 of ‘functional-validated’ proteins know to regulate fitness in response to nitrogen stress or following Torin1 addition to the growth media. The perturbation dataset in which each specific phosphorylation site was identified is highlighted according to the colours in the legend alongside identification of specific examples of specific proteins. Specific information for all sites are listed in table 3.

We have previously conducted a global quantitative fitness profiling of a fission yeast strain bank that harboured deletions of all non-essential genes. This enabled us to identify genes whose loss altered fitness in response to nitrogen stress or Torin1-mediated reduction in TORC1 and TORC2 signalling [40]. Encouragingly, 22 of the targets of TOR and nitrogen signalling (non-cell-cycle-regulated group; electronic supplementary material, data tables S14–S16) identified as harbouring phosphorylation sites that changed in the current study were also identified in the cell fitness assessment. This suggests that fluctuating phosphorylation of these substrates is likely to be functionally important in the cellular response to nitrogen stress and altered TOR signalling (figure 8 and table 3). Importantly, three known regulators of TOR signalling (including Tsc2) were among this functionally validated group alongside a known regulator of autophagy Lvs1 (WDFY3/4) (figure 8 and table 3).

**Table 3.** Functionally validated targets and specific information about their phosphorylation. ‘Non-cell-cycle-regulated’ molecules on which there is one or more sites at which phosphorylation levels fluctuate in response to an insult, for which the corresponding gene deletion strain shows altered fitness upon nitrogen stress or exposure to Torin1. Specific *S. pombe* IDs, gene names, phosphorylation site being regulated, product description and human orthologues are indicated. GO-term analysis has been used to identify the biological function that has been previously assigned to each molecule. Upward, or downward, pointing arrows indicate enhancement or reduction in phosphorylation respectively. The colour code indicates the specific perturbation for which fluctuation in phosphorylation at the site was identified; black, nitrogen stress of wild-type; blue, nitrogen stress of *ssp2::ura4^+^*; red, nitrogen stress of *cdc2.asM17*; green, Torin1 addition to wild-type. Sites identified in nitrogen stress cultures of: ↓ WT ↓ no Cdc2 ↓ AMPK or cultures added ↓ Torin1.

proteins with altered fitness on minimal media or Torin1 that display fluctuating phosphorylation				
systematic ID	gene name	P-site	description	human orthologue
transmembrane transport				
SPCC663.03	pmd1	↓S18	leptomycin transmembrane transporter	ABCB1
SPAC30D11.11	izh3	↓S25	ER membrane, transporter, zinc homeostasis (predicted)	PAQR3
SPBC106.05c	tim11	↓T61	F1-FO ATP synthase subunit E	ATP5ME
SPAC11G7.02	pub1	↓S178	HECT-type ubiquitin-protein ligase E3	NEDD4
chromatin organization				
SPAC664.03	paf1	↑S384, S388	RNA polymerase II-associated Paf1 complex	PAF1
SPBC30B4.04c	sol1	↓S333, S342	SWI/SNF complex	ARID1
SPCC548.05c	dbl5	↑S182, S187, S280	ubiquitin-protein ligase E3, degradation of histones	
TOR signalling				
SPBP18G5.03	toc1	↓S399	Tor complex Tor2 interacting protein 1	
SPBC16E9.13	ksp1	↓S378	serine/threonine protein kinase	
SPAC630.13c	tsc2	↑T1034	tuberin, GTPase activator	TSC2
vesicle-mediated transport				
SPBC21D10.12	hob1	↑S297, S300	BAR adaptor protein Hob1	BIN1
SPBC31F10.16	bch1	↓S445	exomer complex ChAPs family	TTC17
autophagy: SPBC28E12.06c	lvs1	↓S933	beige protein homolog	WDFY3
ribosomes: SPAC31G5.17c	rps1001	↓S135	40S ribosomal protein S10	RPS10
actin: SPCC1919.10c	myo52	↓S798	myosin type V	MYO5
FAD: SPCC1235.04c	nad1	↓S244	FAD synthetase	FLAD1
protein folding: SPBC1711.08	aha1	↓S169	chaperone activator	AHSA1
lysine synthe: SPBC1105.02c	lys4	↑S497	homocitrate synthase	
peroxisomes: SPCC550.09	pex32	↓S198	peroxin Pex32 (predicted)	
vitamins: SPAC1952.08c	SPAC1952.08c	↓T188	pyridoxamine 5'-phosphate oxidase (predicted)	
transcription: SPAC23E2.01	Fep1	↑S231 up ↓S109	iron-sensing transcription factor	
?: SPAC56F8.02	SPAC56F8.02	↑S116	AMP binding enzyme, human DIP2 family (predicted)	DIP2A

### 2.7. Reduced Byr1/Spk1 (MEK/ERK) signalling restrains cell differentiation upon nitrogen stress

**Figure 9.**
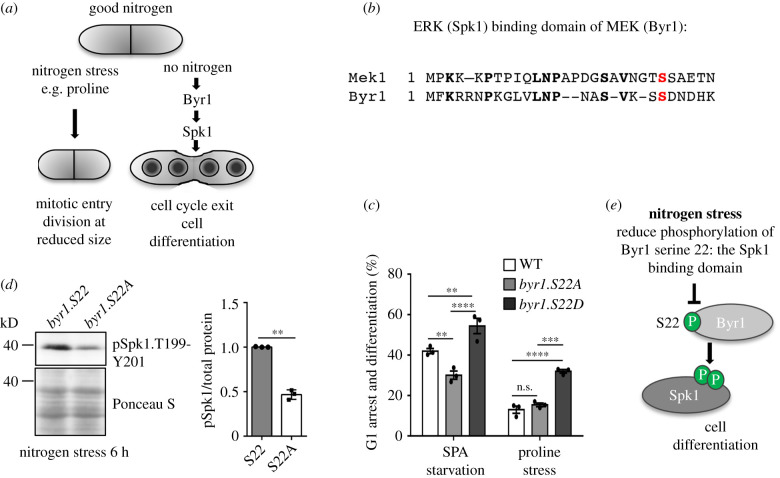
Reduced phosphorylation of the MAPK-binding motif of Byr1 reduce cell differentiation and sporulation. (*a*) A schematic that illustrates the two cell fates a cell can adopt after limitation. Following nitrogen stress (a reduction in nitrogen supply or quality), cells advance mitotic commitment and divide at reduced size. After nitrogen starvation (complete removal of a nitrogen source), cells leave the cell cycle and undergo sexual differentiation meiosis and sporulation. (*b*) An amino acid sequence alignment of *S. pombe* Byr1 and its human orthologue MEK1. Byr1 serine 22 and MEK1 serine 24 are shown in red. (*c*) Biological readout of Byr1 activity for phosphorylation site mutants of Byr1 serine 22 upon nitrogen starvation or nitrogen stress: an analysis of the ability to differentiate to form a zygote with 4 spores after 18 h of either nitrogen stress or nitrogen starvation on agar plates, 500 cells were counted. *n* = 3 with the standard error indicated. **p = 0.01; ***p ≤ 0.001 ****p ≤ 0.0001 (ANOVA, with Tukey's multiple comparison test). (*d*) Cells were grown in MSL to a cell density of 1.8 × 10^6^ cells ml^−1^ and then filtered and resuspended in prewarmed MSL minus nitrogen at same density as the original culture. Western blot analysis of total protein extracts from indicated strains after 6 h of nitrogen starvation. Phosphorylation of the Byr1 substrates Spk1 of threonine 199 and tyrosine 201, function as a biochemical readout of Byr1 MAPK kinase activity. *n* = 3 with the standard error indicated **p *=* 0.01. (Student's *t*-test). Ponceau S staining of the western blot membrane has been included as a loading control. (*e*) Schematic of the role of Byr1.S22 phosphorylation. Phosphorylation at serine 22 is diminished after nitrogen stress in order to stop cells from invoking a full commitment to sexual differentiation until the nitrogen loss is complete.

In contrast with nitrogen stress (change from a rich to a poor nitrogen source), which advances mitotic commitment [5], the nitrogen starvation response to the complete removal of a nitrogen source promotes cell cycle exit and differentiation (figure 9*a*) [41]. Thus, if nitrogen levels or quality is reduced, but not absent, cells remain in the cycle but conserve resources by dividing at reduced size. Importantly, nitrogen stress and nitrogen starvation involve the limitation of the same nutrient, and it is currently unclear how cells distinguish the two scenarios, which have such a dramatically different impact on cell fate.

In fission yeast, pheromone induced Byr2/Byr1/Spk1 MAPK kinase signalling regulates exit from the cell cycle in response to starvation (figure 9*a*) [41]. We were therefore interested to see reduced phosphorylation of a Byr1 peptide that harbours two serines that are conserved in the human orthologue MEK, where their phosphorylation regulates the binding to ERK MAP-kinase to control MAPK signalling [42,43] (figure 9*b*). We therefore investigated the possibility that reduced phosphorylation of the S21/S22 module in Byr1 may represent a mechanism to limit Byr2/Byr1/Spk1 MAPK kinase signalling and cell cycle exit when cells are stressed. In this scenario, reduced phoshophorylation on these sites would actively block the full starvation response to complete nitrogen loss to ensure that cells remain in the cell cycle to await better times, despite the first signs of trouble with a drop in nitrogen levels. In other words, phosphorylation on these sites could be one of the key signalling nodes that differentiates nitrogen stress from nitrogen starvation.

To pursue the exciting possibility that phosphorylation of Byr1 on this peptide differentiates between nitrogen stress and starvation, we mutated the endogenous locus to generate an allelic series of serine point mutants. Phospho-blocking mutants substituted serine for alanine (A) at either site, whereas phosphomimetic mutants substituted serine for aspartic acid (D). Because mass spectrometry was unable to differentiate between phosphorylation on serine 21 or serine 22 (electronic supplementary material, data table S17), we mutated each site independently and assessed the phenotypic impact.

As a readout of Byr1 function, we monitored the impact of altering phosphorylation at either serine 21 or 22 of Byr1 upon the efficiency of sexual differentiation, by monitoring the frequency of mating and subsequent differentiation to produce a zygote with 4 four haploid spores (figure 9*a*). Both phospho-blocking and phosphomimetic mutants of serine 21 (*byr.S21A* and *byr.S21D*) displayed normal mating efficiencies (data not shown). By contrast, *byr1.S22A* and *byr1.S22D* mutations had significant and reciprocal impacts upon the response to 18 h of nitrogen starvation (figure 9*c*). Upon nitrogen starvation, sexual differentiation was significantly increased by phosphomimetic Byr1.S22D, yet reduced by phospho-blocking Byr1.S22A.

To further validate the impact of Byr1.S22 phosphorylation on Byr2/Byr1/Spk1 MAPK kinase signalling and differentiation, we tested the ability of the Byr1.S22D to alter cell fate and promote differentiation following nitrogen stress. Only 10% of wild-type cells undergo differentiation and mating upon chronic nitrogen stress when exposed to the opposite mating partners (growth with proline as a nitrogen source). However, this proportion more than trebled in a *byr1.S22D* mutant background, while the *byr1.S22A* mutant had no impact (figure 9*c*). The similar behaviour of wild-type cells and *byr1.S22A* in nitrogen stresses is entirely consistent with our SILAC phosphoproteomics data, that records a reduction in Byr1.S22 phosphorylation during nitrogen stress.

To further assess the role of Byr1.S22 phosphorylation in MAP kinase signalling, we monitored the ability of Byr1 to promote phosphorylation of the downstream MAP kinase Spk1 after starvation (analogous to MEK control of ERK in humans). In support of the impact of serine 22 phosphorylation status upon cell differentiation (figure 9*c*), the level of activating Spk1 phosphorylation was notably reduced in nitrogen-stressed *byr1.S22A* cells (figure 9*d*).

We conclude that phosphorylation of Byr1 on serine 22 comprises a novel key component in the signalling network that differentiates nitrogen stress from nitrogen starvation. Our data suggest that phosphorylation at this site is diminished to stop cells from invoking a full commitment to sexual differentiation until the nitrogen loss is complete (figure 9*e*). The striking parallels with MEK/ERK signalling validate our approach of exploiting the malleability of yeast genetics to guide the interrogation of nutrient control in higher eukaryotes.

## 3. Discussion

Here, we used quantitative, SILAC and mass spectrometry-based analysis of global TOR and nitrogen-controlled signalling in fission yeast. We exploited the genetic malleability of the system and the cross-species impact of Torin1 to differentiate between distinct networks within larger nitrogen stress and TOR controlled signalling. Further clarity stemmed from the elimination of secondary fluctuations in phosphorylation that occur as a secondary impact of nutrient/TOR control of cell division (figure 1).

We identified more than 1000 sites at which phosphorylation changed upon nitrogen stress or after the addition of Torin1 to block all TOR signalling (figures 2 and 3; electronic supplementary material, data tables S2–S8). One hundred and thirty of these were seen following both perturbations. The identification of peptide, protein and modification site is reported at a false discovery rate (FDR) of 0.01, estimated by the target/decoy approach [44]. Three biological repeats for each experiment would be the ideal number; however, here our data are limited to two repeats for financial reasons, Importantly, our approach is validated by the appearance of more than 80 known effectors of TOR and nitrogen signalling in these datasets (electronic supplementary material, data table S1). Notably, as would be the case for any global studies, the individual sites identified here will require further *in vivo* validation.

A remarkable feature of the datasets is that the frequency at which phosphorylation increased was roughly matched by the frequency with which it declined. It represents a wholesale re-setting of cell homeostasis. Clearly, it is not possible to determine from these data the relative contribution of the regulation of protein kinases and that of protein phosphatases to the overall fluctuations seen; however, changes in the phosphorylation status of the Greatwall kinase Cek1 and its target Igo1 point to an engagement of PP2A-B55. Greatwall kinases phosphorylate Igo1 homologues throughout eukaryotes to generate a poor substrate for PP2A-B55 that attenuates PP2A-B55 function by ‘unfair competition’ [45]. This system has a major bearing on TOR regulation of mitotic control upon the nutrient stress of a switch from glutamate to phenylalanine as a nitrogen source in fission yeast [29] and has been linked to TOR control of cell cycle exit in budding yeast [46]. Indeed, other studies in budding yeast suggest a broader impact or TOR on phosphatase function as Tap42-Sit4 and Tap42-PP2A phosphatases activities increase following TORC1 inhibition upon nutrient stress [47].

The global change to TOR and nitrogen-controlled signalling mapped here was 30 min after the imposition of change, because TORC1 substrates like Maf1 are dephosphorylated after both nutrient stress and Torin1 addition at this time [7,18]. However, nitrogen and TOR signalling are likely to be highly dynamic, with different changes at distinct phases of the response as cells adapt and establish a new level of homeostasis. Indeed, our pilot time-resolved SILAC phosphoproteomic analysis demonstrates that Amk2.S55 dephosphorylation continues after 30 min when TORC2 but not TORC1 is inhibited in a Torin1-resistant TORC1 mutant [18] (figure 4*c*; electronic supplementary material, data table S13). Therefore, further time-resolved phosphoproteome studies comprising rapid and continued sampling is likely to reveal further depth and breadth to the responses, in much the same way as the dissection of the mitotic phosphoproteome by Swaffer *et al*. [19,20].

Within the group we labelled ‘non-cell-cycle’-regulated proteins (not previously known to be regulated in response to Cdc2 (CDK1), Plk1 (Polo) or Ark1 (aurora kinase) inhibition [19,20,31,32]), an excess of 100 of the proteins that are conserved in human cells are novel substrates of TOR of nitrogen signalling (electronic supplementary material, tables S2–S4). Of these, 74 were regulated by nitrogen stress, 18 by both nitrogen and Torin1 and 25 by Torin1. The human orthologues regulate a wide variety of biological processes that are known to be modified by TOR and nitrogen signalling (tables 4–6). Importantly, we have previously validated the functional relevance of several sites identified here. This includes the site on fission yeast CAPZA (electronic supplementary material, data table S4) as a target of TORC2 [48]. We have shown how the SAGA complex that incorporates the conserved TAF10 component identified as a target in our study (electronic supplementary material, data table S2) is phosphorylated in response to nitrogen starvation [35], and we have shown that the novel site on Gad8 (AKT) serine 93 (electronic supplementary material, data table S8) within the PM binding domain of the kinase, is required for nutrient control of cell division [15].

**Table 4.** Targets conserved in human cells not previous associated with nitrogen signalling. A list of human orthologues of the new targets of nitrogen signalling. GO-term mapping indicated the biological process regulated by the individual proteins.

novel nitrogen-regulated proteins—human orthologues
RNA metabolic processes *(including: ribosome biogenesis, splicing, transcription, RNA catabolic process)*
ABT1 CNOT1 CTNNBL1 CTR9 DDX17 DDX49 ESF1 IVL MED11 MTREX NIFK PLRG1 PPP2R2A RBM22 RNF10 RPAP1 RPL28 RPL35A RPS10 SCAF8 SREBF1 SRSF2 TAF10 TAF2 TAF9B THOC2 TRPT1 TTF1 UTP3
transport *(including vesicle-mediated transport, transmembrane transport)*
ABCB1 ARG1 ARID1A ATP13A1 ATP8A1 BIN1 BMP2 K CANX ESYT1 HID1 MAPK14 PDPK1 PSMD11 PSTPIP1 TAF7 TBC1D22A TRAPPC9 TTC17 WAS
chromosome organization
ARID1A CDC45 MORF4 SETD6 SIRT2 TAF7 USP7
lipid metabolic process
CERS2 ESYT1 GPCPD1 ORMDL1 PPM1 L
signal transduction
PPML1 PPP1R2 SUB1 URI1 ZNF622
autophagy
ATP6V1G1 CAMKK2 HSP90AA1 TSC2
mitochondrial ATP synthesis-coupled proton transport
ATP5ME
electron transfer activity
CIAPIN1
FAD biosynthetic process
FLAD1
protein folding
HSPA14
arginine biosynthetic process via ornithine
ASL

**Table 5.** Targets conserved in human cells not previous associated with both nitrogen and Torin1-regulated signalling. A list of human orthologues of the new targets of nitrogen and Torin1 signalling. GO-term mapping indicated the biological process regulated by the individual proteins.

novel nitrogen and Torin1-regulated proteins—human orthologues
RNA metabolic processes*(including: splicing, ribosomal protein, RNA stability)*
DYRK1A RPS3A SCAF8 SNW1 SRSF5 TCF25
translation
EIF2B5 ETF1 CNOT7 SAMD4B UPF1
regulation of DNA repair
Trip12
vitamin metabolic process
GCH1
signal transduction
PPP2R5A
autophagy
NSFL1C
haem biosynthetic pathway
ALAS1
polarized cell growth
LRRC57
amino acid metabolic process (serine)
PHGDH

**Table 6.** Targets conserved in human cells not previous associated with Torin1-regulated signalling. A list of human orthologues of the new targets of Torin1 signalling. GO-term mapping indicated the biological process regulated by the individual proteins.

novel Torin1-regulated proteins—human orthologues
RNA metabolic processes *(including: translation, splicing, ribosomal protein, RNA stability)*
CSTF2T POLR3B RPAP1 RPS23 TARS1 TRNAU1AP UPF2 ZCCHC7
vesicle-mediated transport
ARFGAP1 EPN3 RIC1 SEC31A UNC13A
cytoskeleton organization
CAPZA2 NEBL PI4KA PLEK2 KIAA1217
autophagy
WDFY4
protein folding
AHSA1
metal ion homeostasis
PAQR3
mitochondrial ATP synthesis
ATP5F1D
ER protein translocation
SEC63
Hippo signalling pathway
MOB1A
ER stress and unfolded protein response
ERMP1

Overall, most of the novel targets identified are involved with RNA metabolic processes (including ribosome biogenesis, splicing, transcription, translation, RNA stability). Forty-eight of the novel human orthologues (40%) belong to this group (tables 4–6), supporting the well-established role of gene expression as a major means by which cells adapt to nutrient shortage and TOR inhibition. Several of the targets identified as having altered phospho-status following nitrogen stress regulate transmembrane and vesicle-mediated transport, both of which are known to enhance nutrient uptake. In our Torin1 response dataset, the potential for the modulation of translocation into the endoplasmic reticulum by Sec63 is of interest (table 6). To our knowledge, this is the first time this element of membrane traffic has been associated with TOR signalling.

In response to nitrogen stress, we find that the MAPKK Byr1 is dephosphorylated within the MAPK kinase docking site at which it engages with Spk1. This is likely to represent a significant new component in the signalling network that restrains cell cycle exit and sexual differentiation when resources are sparse (figure 9*e*). This highlights the delicate balance struck when nutrients start to get sparse. Upon nitrogen stress, cells reduce their energy requirements to maintain proliferation since in the longer term, this will be beneficial. When nitrogen is completely removed, the cell must act quickly to use the limited reserves of nitrogen it is composed of. Cell differentiation, mating, meiosis, sporulation followed by re-germination of spores are more energy-consuming than simple reducing growth and size. However, undergoing meiosis ‘reshuffles’ the genome through meiotic recombination that could generate a novel genotype that may be able to accommodate the changes in the compromised environment.

In mammalian as well as budding yeast cells, scaffolding proteins, which act to coordinate the assembly of kinase-kinase complexes, regulate MAPK signalling. An example of such a scaffold protein for the Ras–Raf–MEK pathway is the Kinase suppressor of Ras 2 (KSR2), which controls the activity of MEK in a range of organisms [43]. A crystal structure of the human KSR2 kinase domain in complex with rabbit MEK1 has demonstrated that the serine 24 (corresponding to Byr1.S22; figure 9*b*) is phosphorylated in a KSR2-dependent manner [43]. The equivalent scaffolding proteins in fission yeast are currently unknown. In an effort to identify the kinase responsible for Byr1 S22 phosphorylation, we attempted to generate phospho-specific antibodies; however, unfortunately, this was unsuccessful. Thus, at this stage, we are unable to define the kinase responsible for modulation of Byr1 serine 22 phosphorylation. However, our data do highlight the ubiquity of phospho-regulation of this protein-binding in response to environmental changes.

Consistent with the general level of protein conservation from yeast to humans, 70% of the substrates identified here are conserved in human cells (electronic supplementary material, data table S9). This conservation highlights the utility of drawing upon these analyses in yeast as a highly controlled model system to guide the interrogation of TOR signalling in higher eukaryotes [18].

## 4. Conclusion

This analysis provides a rich source of novel and conserved potential substrates of TOR and nitrogen signalling (table 4–6) that regulate a diverse spectrum of biological processes to modulate growth and proliferation in response to environmental stress.

## 5. Material and methods

### 5.1. Yeast cell cultures used for SILAC and mass spectrometry

Strains used in this study are *car2::NAT lys1–131 arg3-d4* and derivatives incorporating *ssp2::ura4+ or cdc2.asM17 or tor2.G2040D* mutant alleles (laboratory stock)*.* All cultures were grown at 28°C. Cells were inoculated in YES medium overnight [49] and then washed into Edinburgh minimal media (EMM2-N) (ForMedium) [50] supplemented with 20 mM l-Glutamic acid (EMMG) [49] and 75 mg l^−1^ of either light [l-arginine monohydrochloride (Sigma) and l-lysine monohydrochloride (Sigma)] or medium [lysine-l, 2HCl 4.4.5.5-D4 (Cat code DLM-2640, Eurisotop), arginine-L, HCl, U-13C6 99%13C (cat. no. CLM-2265, Eurisotop)] amino acids. Cells were cultured in the log phase for 48 h to ensure complete incorporation of labelled amino acids into the proteome.

Torin1 treatments: light-labelled cultures were treated with DMSO and medium-labelled cultures were treated with a final concentration of 25 µM Torin1 at a density of 2.04 × 10^6^ cells ml^−1^.

Nitrogen stress: light-labelled cultures were filtered into EMMG containing 75 mg l^−1^ of light [l-arginine monohydrochloride (Sigma) and l-lysine monohydrochloride (Sigma)]. Medium-labelled cultures were filtered into Edinburgh minimal media (EMM2-N) [50] containing 20 mM L-Proline (EMMP) [49] at a density of 2.5 × 10^6^ cells ml^−1^.

Cdc2 inhibition: light-labelled cultures were filtered into EMMG containing 75 mg l^−1^ of light [l-arginine monohydrochloride (Sigma) and l-lysine monohydrochloride (Sigma)]. Medium-labelled cultures filtered into EMMP containing 20 µM 3BrB-PP1 (3-[(3-Bromophenyl)methyl]-1-(1,1-dimethylethyl)-1H-pyrazolo[3,4-d]pyrimidin-4-amine, Toronto Research Chemicals) at a density of 2.5 × 10^6^ cells ml^−1^.

Approximately 5 × 10^9^ cells were harvested for each sample. After 30 min, cultures were harvested by centrifugation (3000*g* for 5 min), washed in 20 ml of STOP buffer (10 mM EDTA, 1 mM sodium azide, 50 mM sodium fluoride, 0.9% NaCl), followed by washing with 10 ml of ice-cold ddH_2_O. The final pellets were then resuspended in an appropriate volume of ice-cold ddH_2_O and dropped directly into liquid nitrogen to produce frozen cell droplets.

### 5.2. SILAC protein extraction

Samples were processed using a SPEX Sample Prep LLC 6850 Freezer Mill in the presence of liquid nitrogen. The resulting cell powder was resuspended in denaturation buffer (6 M urea, 2 M thiourea, 1% *n*-octyl glucoside) at a ratio of 500 mg powder to 500 µl denaturation buffer. Insoluble material was removed by centrifugation (13 000*g*, 10 min at 4°C) and the supernatant was designated supernatant I (soluble fraction). The pellet was then resuspended in 500 µl denaturation buffer, 500 µl glass beads were added and then subjected to 20 s shaking in a FastPrep machine (FP120, Qbiogene). The resulting suspension was again centrifuged (13 000*g*, 10 min at 4°C) and the supernatant retained (supernatant II). The pellet was then discarded. Protein concentrations were determined by Bradford assay according to the manufacturer's instructions.

### 5.3. Mass spectrometry for SILAC

Respective supernatants I and II derived from the ‘light’ and ‘medium’ labelled cell cultures were combined and proteins were precipitated at −20°C using ice-cold acetone in methanol left on ice overnight. The proteins were pelleted by centrifugation (2200*g*, 20 min, 4°C) and washed with 80% ice-cold acetone. Dried proteins were resolved in digestion buffer (6 M urea, 2 M thiourea, 10 mM Tris–HCl, pH 8.0) and mixed at a 1 : 1 ratio according to measured protein amounts. The mixtures were digested in solution with trypsin as described previously [51].

For proteome analyses of wild-type cells and *ssp2::ura4^+^*, 100 µg of the mixtures was fractionated by isoelectric focusing on an OffGel 3100 Fractionator (Agilent) according to the manufacturer's instructions. Focusing was performed using 13 cm (12 well) Immobiline DryStrips pH 3–10 (Bio-Rad) at a maximum current of 50 µA for 24 kVh. Peptide fractions were collected and desalted separately using C18 StageTips [52].

For phosphoproteome analyses of extracts from Torin1 treatment, nitrogen stress of wild-type cells and nitrogen stress *ssp2::ura4^+^*: 8 mg of each peptide mixture was subjected to phosphopeptide enrichment as described previously [53] with minor modifications: peptides were separated by strong cation-exchange (SCX) chromatography with a gradient of 0–35% SCX solvent B resulting in seven fractions that were subjected to phosphopeptide enrichment by TiO_2_ beads. Elution from the beads was performed three times with 100 µl of 40% ammonia hydroxide solution in 60% acetonitrile (pH > 10.5). Fractions rich in peptides were subjected to multiple TiO_2_ enrichment. Enrichment of phosphopeptides from the SCX flow-through was completed in five cycles.

Liquid chromatography tandem mass spectrometry (LC-MS/MS) analyses were performed on an EasyLC nano-HPLC (Proxeon Biosystems) coupled to an LTQ Orbitrap XL (Thermo Scientific) for phosphopeptide analyses, or an LTQ Orbitrap Elite mass spectrometer (Thermo Scientific) for proteome analyses as described previously [31]. The peptide mixtures were injected onto the column in HPLC solvent A (0.5% acetic acid) at a flow rate of 500 nl min^−1^ and subsequently eluted with a 87 min (proteome) or a 127 min (phosphoproteome) segmented gradient of 5%, 33% and 90% HPLC solvent B (80% acrylonitrile in 0.5% acetic acid). During peptide elution, the flow rate was kept constant at 200 nl min^−1^. For proteome analysis, the 20 most intense precursor ions were sequentially fragmented in each scan cycle. For the phosphoproteome analysis, the five most intense precursor ions were fragmented by multistage activation of neutral loss ions at −98, −49 and −32.6 Th relative to the precursor ion [54]. In all measurements, sequenced precursor masses were excluded from further selection for 90 s. Full scans were acquired at a resolution of 60 000 (Orbitrap XL), or 120 000 (Orbitrap Elite). The target values were set to 5000 charges for the LTQ (MS/MS) and 10^6^ charges for the Orbitrap (MS), respectively; the maximum allowed fill times were 150 ms (LTQ) and 1000 ms (Orbitrap). The lock mass option was used for real-time recalibration of mass spectrometry spectra [55]. The mass spectrometry data of all SILAC experiments were processed using default parameters of the MaxQuant software (v. 1.2.2.9) [56]. Extracted peak lists were submitted to database search using the Andromeda search engine [44] to query a target–decoy database of *S. pombe* proteome (http://www.pombase.org/, Protein Dataset in FASTA format, downloaded on 6 April 2011), containing 5076 protein entries and 248 commonly observed contaminants.

In the database search, full tryptic specificity was required and up to two missed cleavages was allowed. Carbamidomethylation of cysteine was set as fixed modification; protein N-terminal acetylation, oxidation of methionine, phosphorylation of serine, threonine, and tyrosine, and Arg6 → Pro5 substitution (to address the arginine-to-proline conversion effect) were set as variable modifications. Initial precursor mass tolerance was set to 6 ppm at the precursor ion and 0.5 Da at the fragment ion level. FDRs were set to 1% at peptide, phosphorylation site and protein group level. For protein group quantitation, a minimum of two quantified non-phosphorylated peptides were required, for phosphorylation sites at least one quantitation event was required. Quantified phosphorylation sites were further normalized for changes on the proteome level by dividing the site ratio by the corresponding protein group ratio using R v. 2.15.0 as described previously [57].

For phosphoproteome analyses of extracts from nitrogen-stressed *cdc2.asM17:* 20 µg of the resulting peptides was directly desalted with C_18_ StageTips [52] and further analysed on an Easy-nLC system coupled to a Q Exactive HF mass spectrometer (both Thermo Fisher Scientific). Eight milligrams of peptide mixture were purified on a Sep-Pak 18 cartridge (Waters). Briefly, the cartridge was activated with 5 ml methanol, conditioned with 5 ml of 1% trifluoroacetic acid (TFA)/2% acetonitrile (ACN) and subsequently loaded with the sample. Peptides were washed with 5 ml of 0.5% acetic acid and eluted with 5 ml 6% TFA in 80% ACN. Phosphopeptide enrichment was done using TiO_2_ beads (Zirchrom Separations Inc.) that were washed twice in loading solution (6% TFA in 80% ACN) and added to the sample in a peptide to bead ratio of 2 : 1 (mg mg^−1^). After an incubation time of 10 min, beads were spun down. The supernatant was subsequently used for further sequential enrichment for a total of 10 enrichment rounds. Beads loaded with peptides were washed three times with 150 µl 0.5% TFA in 50% ACN. TiO_2_ beads were resuspended in 200 µl 0.5% TFA in 80% ACN and transferred to a C_8_ StageTip. Elution from the beads was performed two times with 100 µl of 5% ammonia hydroxide solution (pH11), followed by 50 µl 1% formic acid (FA) in 80% ACN.

LC-MS/MS analyses were performed as described elsewhere [58] with slight modifications: 227 min (proteome) or 87 min (phosphoproteome) segmented gradient of 10%, 33%, 50% and 90% of HPLC solvent B (80% acetonitrile in 0.1% formic acid) in HPLC solvent A (0.1% formic acid) was applied at a flow rate of 200 nl min^−1^. For proteome analysis, the 12 most intense precursor ions, and for the phosphoproteome analysis, the 7 most intense precursor ions were sequentially fragmented in each scan cycle using higher energy collisional dissociation (HCD) fragmentation. In all measurements, sequenced precursor masses were excluded from further selection for 30 s. Full scans were acquired at a resolution of 120 000. The target values were set to 10^5^ charges for the MS/MS fragmentation and 3 × 10^6^ charges for the MS scan, respectively. The mass spectrometry data of all SILAC experiments were processed using default parameters of the MaxQuant software (v. 1.5.2.8) [56]. Extracted peak lists were submitted to database search using the Andromeda search engine [44] to query a target–decoy [59] database of *S. pombe* proteome (http://www.pombase.org/, Protein Dataset in FASTA format, downloaded on 6 April 2011), containing 5076 protein entries and 285 commonly observed contaminants. Endoprotease trypsin was defined as protease with a maximum of two missed cleavages. The oxidation of methionine, phosphorylation of serine, threonine, and tyrosine, and N-terminal acetylation were specified as variable modifications, whereas carbamidomethylation on cysteine was set as fixed modification. The initial maximum allowed mass tolerance was set to 4.5 parts per million (ppm) for precursor ions and 20 ppm for fragment ions. Peptide, protein and modification site identifications were reported at an FDR of 0.01, estimated by the target/decoy approach [44]. For protein group quantitation, a minimum of two quantified peptides were required. To assign a P-site to a specific residue, we used a minimal reported localization probability of 0.75, and we refer to these as localized phosphorylation sites. The detected phosphorylation sites were normalized for changes in protein abundance using R v. 3.5.1 [57].

Pilot time-resolved SILAC mass spec analysis of samples processed using a SPEX Sample Prep LLC 6850 Freezer Mill in the presence of liquid nitrogen were performed as described previously [60]. Data were analysed with MaxQuant [56] (v. 1.6.0.9) using the Andromeda search engine [44] to query a target–decoy database of *S. pombe* from UniProt (September 2019 release).

### 5.4. Cell division ratio measurements

For cell division index, cells were fixed with 37% formaldehyde (final 10% v/v) and washed with 1 × PBS. Septa were stained with calcofluor white [61] (Sigma-Aldrich). Dividing cells were counted (greater than 200 cells counted per time point). Images of cells were obtained using a microscope and CoolSNAP HQ2 CCD camera and processed with ImageJ.

### 5.5. Yeast cell cultures used to study Byr1 S22 function

Endogenous Byr1.S22 mutant strains have been prepared using standard molecular biology techniques and the *natMX6/rpl42^+^* cassette for positive and negative selection [62]. All cultures were grown in log phase for 48 h in MSL at 28°C [49]. To impose nitrogen starvation in liquid cultures, cells at a cell density of 1.8 × 10^6^ cells ml^−1^ were filtered into MSL—nitrogen [49] cells were harvested filtration and snap frozen in liquid nitrogen.

For all sexual differentiation assays: two cultures of opposite mating types both harbouring the same mutation in Byr1.S22 were grown in MSL to a cell density of 1.8 × 10^6^ cells ml^−1^. The cultures of opposite mating types were mixed 1 : 1 before being filtered and spotted onto SPA (nitrogen starvation) or EMMP (nitrogen stress) agar plates [49]. After 18 h, cells were scraped off the agar plates and resuspended in H_2_O. Five hundred cells were counted for each assay. Mating frequencies were calculated as described previously [63].

### 5.6. Western blotting

TCA precipitation protocol was followed for *S. pombe* total protein extracts [64]. The following dilutions of antibodies were used in this study: mouse TAT1 [65] (1 : 2000; Kind gift from K. Gull), mouse PK-Tag (1 : 2000; AbD Serotec, Oxford). Anti-phospho-ERK1/2 (pThr^202/Tyr204^) (SAB4301578 SIGMA) was used to detect Spk1 phosphorylation. Alkaline phosphatase-coupled secondary antibodies were used for all blots followed by direct detection with NBT/BCIP (VWR) substrates on PVDF membranes.

## Data accessibility

This article has no additional data.
